# Hand osteoarthritis phenotypes based on a biopsychosocial approach, and their associations with cross-sectional and longitudinal pain

**DOI:** 10.1016/j.joca.2024.04.011

**Published:** 2024-04-30

**Authors:** Elisabeth Mulrooney, Tuhina Neogi, Hanne Dagfinrud, Hilde B. Hammer, Pernille S. Pettersen, Tore K. Kvien, Karin Magnusson, Ida K. Haugen

**Affiliations:** # Center for Treatment of Rheumatic and Musculoskeletal Diseases (REMEDY), Diakonhjemmet Hospital, Oslo, Norway; † Faculty of Medicine, University of Oslo, Oslo, Norway; ‡ Section of Rheumatology, Boston University School of Medicine, Boston, United States; § Lund University, Faculty of Medicine, Department of Clinical Sciences Lund, Orthopaedics, Clinical Epidemiology Unit, Lund, Sweden

**Keywords:** Osteoarthritis, Hand osteoarthritis, Pain, Pain sensitization, Phenotypes

## Abstract

**Objective:**

Hand osteoarthritis (OA) pain is characterized as heterogeneous and multifactorial. Differences in pain may be explained by underlying phenotypes, which have not been previously explored

**Design:**

Latent class analysis determined classes of participants with hand OA from the Nor-Hand study baseline examination (2016–17) based on a biopsychosocial framework. Outcomes were hand and overall bodily pain intensity (Numeric Rating Scale, 0–10) at baseline and follow-up (2019–21), The relations of the classes to pain outcomes at baseline, follow-up, and change over time were analysed in separate models by linear regression, using the overall healthiest class as reference.

**Results:**

Five classes differing in radiographic hand OA burden and OA burden in the lower extremities by ultrasound, demographic factors, psychosocial burden and pain sensitization was identified. Persons with the least severe OA but higher burden of biopsychosocial factors reported the most hand pain (beta 3.65, 95% CI 2.53, 4.75). Pain was less pronounced in persons with the most severe hand OA but low burden of biopsychosocial factors (beta 1.03, 95% CI 0.41, 1.65). Results were similar for overall bodily pain and at follow-up. Changes in pain were small, but the association between a separate class defined by higher levels of biopsychosocial burden and pain changes was significant.

**Conclusion:**

The five hand OA phenotypes were associated with pain at baseline and 3.5 years later. The phenotype with the least OA severity, but higher burden of biopsychosocial factors reported more pain than the phenotype with the most severe OA, reflecting the symptom-structure discordance of the hand OA pain experience.

## Introduction

Pain in OA is a complex phenomenon, and has significant impact on quality of life.^1^ Traditionally, OA pain was thought to originate from structural joint damage and/or inflammation. However, there is a discordance between the burden of symptoms and joint pathology.^2–4^ The pain in hand osteoarthritis (OA) is both heterogeneous and multifactorial^5,6^ and can involve biological, psychological and social aspects extending beyond OA joint pathology. Pain in people with OA are cross-sectionally related to factors representing the biological domain such as pain sensitization, overweight/obesity and comorbidities, factors of the social domain such as emotional and cognitive factors, and factors reflecting the social domain like socioeconomical status.^7–10^ Currently, we have limited knowledge about the pain experience in people with hand OA in the context of a biopsychosocial framework.

Considering the multifactorial and heterogenous nature of OA,^5,6^ the hand OA pain experience may be due to subtypes or possible phenotypes determined by a co-occurrence of factors across multiple domains. A phenotype has been defined as “Any observable characteristic or trait of a disease, such as morphology, development, biochemical or physiologic properties, or behavior, without any implication of a molecular mechanism or pathway”.^11^ There is a lack of studies focused on investigating phenotypes of OA in hands among other less studied joints.^12^ In terms of hand OA, thumb base OA vs. interphalangeal OA and non-erosive vs. erosive hand OA have been suggested as different phenotypes.^13,14^ Possible hand OA phenotypes based on factors beyond joint pathology have not previously been explored. Therefore, we aimed to identify possible hand OA phenotypes using the biopsychosocial model of pain as a framework and explore their associations with pain severity and change in pain during a 3,5-year-follow-up.

## Methods

### Patients and study design

These longitudinal analyses are based on data collected at the baseline examination in 2016–17 and the follow-up in 2019–2021 of the Nor-Hand study, a hospital-based observational cohort study following 300 participants with hand OA. Men and women between 40–70 years with hand OA were consecutively recruited from the Rheumatology outpatient clinic at Diakonhjemmet Hospital, and included if OA was present in at least one finger or thumb base joint confirmed by ultrasound and/or clinical examination. Participants were excluded if they had rheumatoid arthritis, spondylarthritis, psoriatic arthritis, psoriasis, or hemochromatosis. Ahead of the follow-up examination, the participants were asked about development of systemic inflammatory joint diseases and skin psoriasis and excluded in case of development of such diseases. Detailed inclusion and exclusion criteria are described in previously published protocols.^15,16^ The study was approved by the Norwegian Regional Committee for Medical and Health Research Ethics (Ref. no: 2014/2057 and 2019/363) and registered at https://clinicaltrials.gov (Ref. no: NCT03083548). The participants received oral and written information about the study and provided their informed consent. They were informed that they could withdraw at any time throughout the study. A patient partner was involved during study planning and throughout the study period, and contributed with input on the study design, interpreting and disseminating results.

### Hand OA phenotypes based on biological, psychological, and social factors

**Fig. 1:**
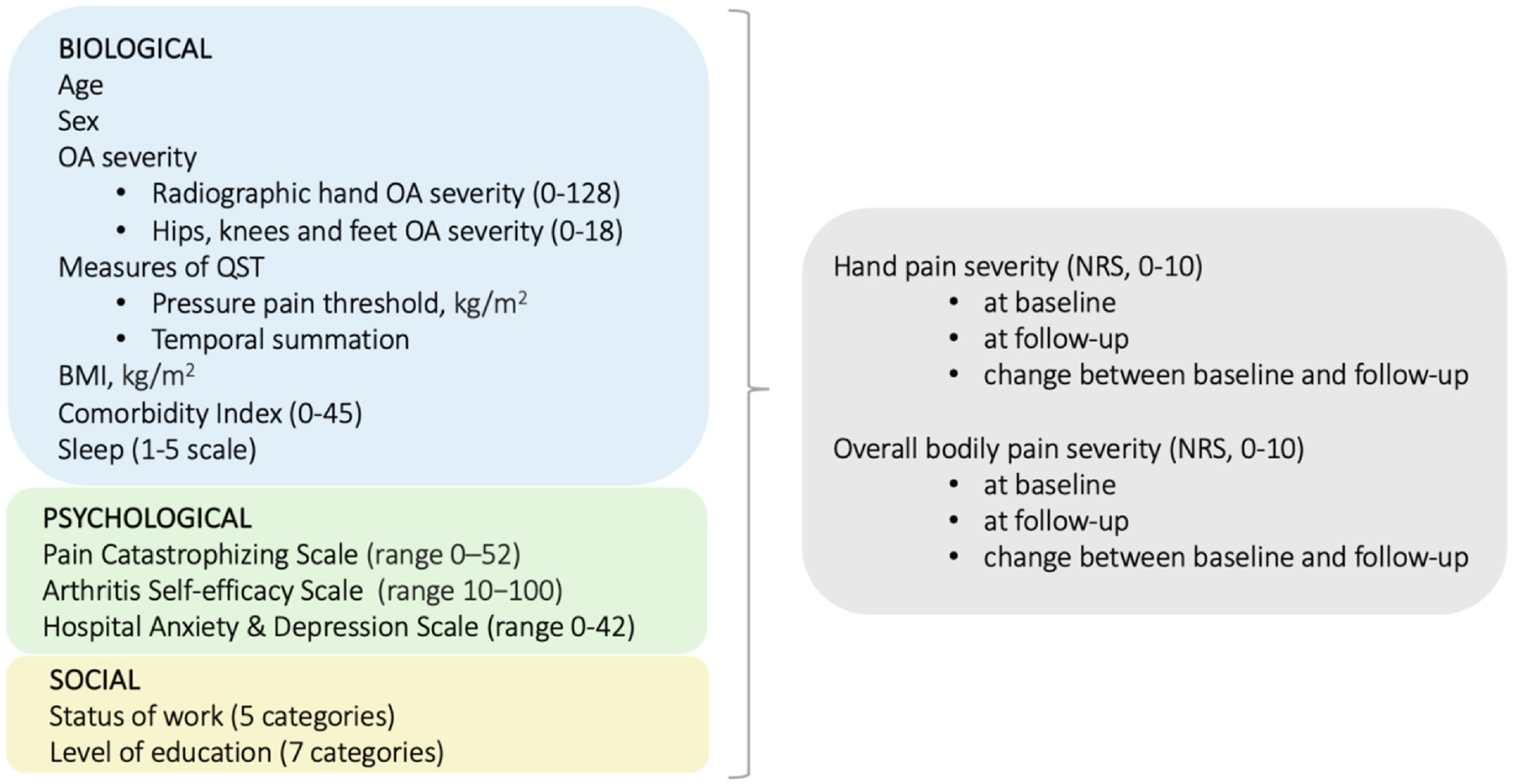
The biopsychosocial variables used for defining classes, and their outcome measures.

To determine the hand OA phenotypes, we included factors from biological, psychological, and social domains, as outlined below (Fig. 1).

For the biological domain, information about age and sex was collected from medical records. Height (without shoes) and weight (in light clothing) was measured by medical students and BMI was calculated (kg/m^2^). Radiographic hand OA severity was determined by a trained reader (IKH) according to a modified Kellgren-Lawrence score (0–4).^17^ A sum score of all hand joints was calculated, including distal and proximal interphalangeal, metacarpophalangeal, first carpometacarpal and scaphotrapeziotrapezoid joints (range: 0–128). The intra-observer reliability for Kellgren-Lawrence sum score was 0.99 (n = 20 randomly selected radiographs scored with a mean (standard deviation) of 16 (4) days between first and second scoring). OA of the hips, knees and feet was calculated as a sum score of ultrasound-detected osteophytes (each joint scored on a 0–3 scale) based on an ultrasound examination performed by two trained medical students.^15^ The osteophyte sum score included both hips, maximum score of four compartments in each knee and highest score of all joints in each ankle/foot (range: 0–18). Inter-observer reliability (intraclass correlation coefficient (ICC), two-way mixed-effects model, absolute agreement, individual measure) for the sum score of ultrasound-detected osteophytes in the hips, knees, and feet (n = 10 participants) was 0.82.

Quantitative sensory testing (QST) is a tool to indirectly assess central pain sensitization. Two trained medical students assessed pressure pain detection threshold (PPT) (kg/cm^2^), which is the point a sensation from pressure is first experienced as painful, and temporal summation (TS), which is defined as an increase in pain intensity during the repetition of identical noxious stimuli (i.e., ascending nociceptive facilitation). PPT was tested at the mid-portions of tibialis anterior muscle using a hand-held algometer (FPIX25 Wagner; Wagner Instruments, Greenwich, USA) and TS was assessed at the left distal radioulnar joint, according to a published protocol.^15^ Nine subjects were examined by both examiners the same afternoon. Moderate inter-observer reliability (ICC, two-way mixed-effects model, absolute agreement, individual measure) was 0.43 for PPT at the tibialis anterior and 0.56 for TS.

To assess the comorbidity burden, participants reported their comorbidities at baseline using a Comorbidity index,^18^ which was modified by removing one of the pre-defined medical conditions of the 12 in the original index. The comorbidity “OA” was removed, as all participants had hand OA and we could not conclude whether the question was answered according to OA in other joints. The modified index includes 11 medical conditions (heart disease, high blood pressure, lung disease, diabetes, ulcer or stomach disease, kidney disease, liver disease, anemia/other blood disease, cancer, depression, and back pain) and three optional conditions. For each comorbidity, the participants were asked to indicate whether he/she had the disease, and if so, whether he/she received treatment for the disease and whether the disease was causing limitations. Higher scores indicate higher burden (range of sum score: 0–42). Sleep disturbance was self-reported according to five statements; normal sleep (no problems), slight problems (e.g. difficulty in falling asleep, or sometimes waking at night), moderate problems (e.g. disturbed sleep, or feeling I have not slept enough), greater problems (e.g. having to use sleeping pills often or routinely, or usually waking at night and/or too early in the morning) or severe sleeplessness (e.g. sleep is almost impossible even with full use of sleeping pills, or staying awake most of the night).^19^ We reported the proportion of participant that had slight to severe sleep disturbance.

The psychological domain was assessed by three questionnaires completed by the participants. Hospital Anxiety and Depression Scale (HADS, range: 0–42) includes seven questions regarding anxiety and seven questions regarding depression.^20^ Pain catastrophizing was assessed the Pain Catastrophizing Scale (PCS, scale: 0–52).^21^ Higher scores for HADS and PCS questionnaires indicate worse status. Self-efficacy was examined by The Arthritis Self Efficacy scale (ASES, scale: 10–100), originally designed for rheumatoid arthritis.^22^ One part (5 questions) relates to the ability to influence pain, and the other part (6 questions) relates to the ability to influence other symptoms of rheumatic disease. Higher score indicates greater self-efficacy.

For the social domain, education and work status was assessed as a proxy for socioeconomic status. Education was reported according to the highest level of completed education, consisting of seven levels ranging from 7 years elementary school or shorter to four years or more of university/higher education. Participants reported their status of work according to whether they were currently working, on sick leave, on rehabilitation benefit, disability benefit, unemployed or retired. We have reported the proportion of participants that had higher education and who were currently working.

## Outcomes

### Self-reported pain severity

Severity of pain in the hands during the last 24 h was self-reported on the Numeric Rating Scale (NRS, range: 0–10) at both baseline and at the 3.5-year-follow-up. As an additional outcome and a proxy of central sensitization in people with hand OA, NRS overall pain severity the last 24 h (0–10) at baseline and follow-up was included. Change in pain between baseline and follow-up for both pain measures were calculated as the difference between the two values (baseline value subtracted from the follow-up value).

### Statistical analysis

To identify potential hand OA phenotypes, we applied a mixed-model Latent Class Analysis (LCA) to detect classes of participants based on latent biopsychosocial similarities. Models with an increasing number of classes were estimated until the best-fitting model was observed (two to six classes). All participants were included in the LCA as none were missing > 4 indicator variables at baseline. A minimum class size of 5% of participants was imposed.^23^ We used posterior fit statistics of Bayesian Information Criterion (BIC), Akaike Information Criterion (AIC) and Bootstrap Likelihood ratio Test to assess goodness of fit of the models, and determine the optimal number of classes. For each of these, the model with the lowest goodness of fit value indicates the optimal number of classes. Entropy was calculated to assess the precision for assigning the participants to their most likely class. In addition to the fit-criteria, the model was also considered in the context of current knowledge in order to select the model that best represented the data. Once we identified the optimal number of classes, the probability of membership in each latent class was estimated. We assigned the participants to the class corresponding to their maximum posterior probability. Participant characteristics were presented according to class membership. One-way ANOVAs for continuous variables and Chi square tests for categorical variables were used for to assess differences of the biopsychosocial factors between the classes. To determine which classes differed, a post-hoc test by Bonferroni was applied. Linear regression analyses were used to examine the associations between classes and pain outcomes. Missing data were < 2% for the pain outcomes, and regression analyses were thus performed as a complete case analysis. All analyses were performed with Stata/IC 16.1.

## Results

At the baseline examination, 300 participants were included in the Nor-Hand study. After a mean follow-up time of 3.5 years (range: 2.4–4.2 years), 87 persons (27%) were lost to follow-up due to un-willingness to participate (n = 56), not available for contact (n = 27) or development of systemic inflammatory joint diseases and/or psoriasis (n = 4). All 300 participants were included in the LCA analysis, whilst the 213 participants who attended both examinations were included in analyses utilizing on follow-up data. The baseline characteristics across indicator variables and the pain outcomes were similar when comparing participants lost to follow-up and the study population included in the study (Supplementary Table I).

We ran LCA models starting with two classes. The model with six classes resulted in one of the classes including less than 5% of the participants. Of the remaining models (two to five classes), the model with five classes displayed the most favorable Bootstrap Likelihood Ratio Test, AIC and BIC values. Higher entropy closer to 1 suggests that classes do not overlap and are distinct, and entropy for the five-class model was 0.90.^24^ The 5-class model was also the most clinically interpretable based on our theoretical understanding. The average posterior probability of class membership for each class ranged between 0.87 and 0.95. Values above 0.90 are considered ideal, however all average latent class posterior probabilities are not required reach the > 0.90 criterion if the model is theoretically supported and other criteria are met.^23^

**Fig. 2:**
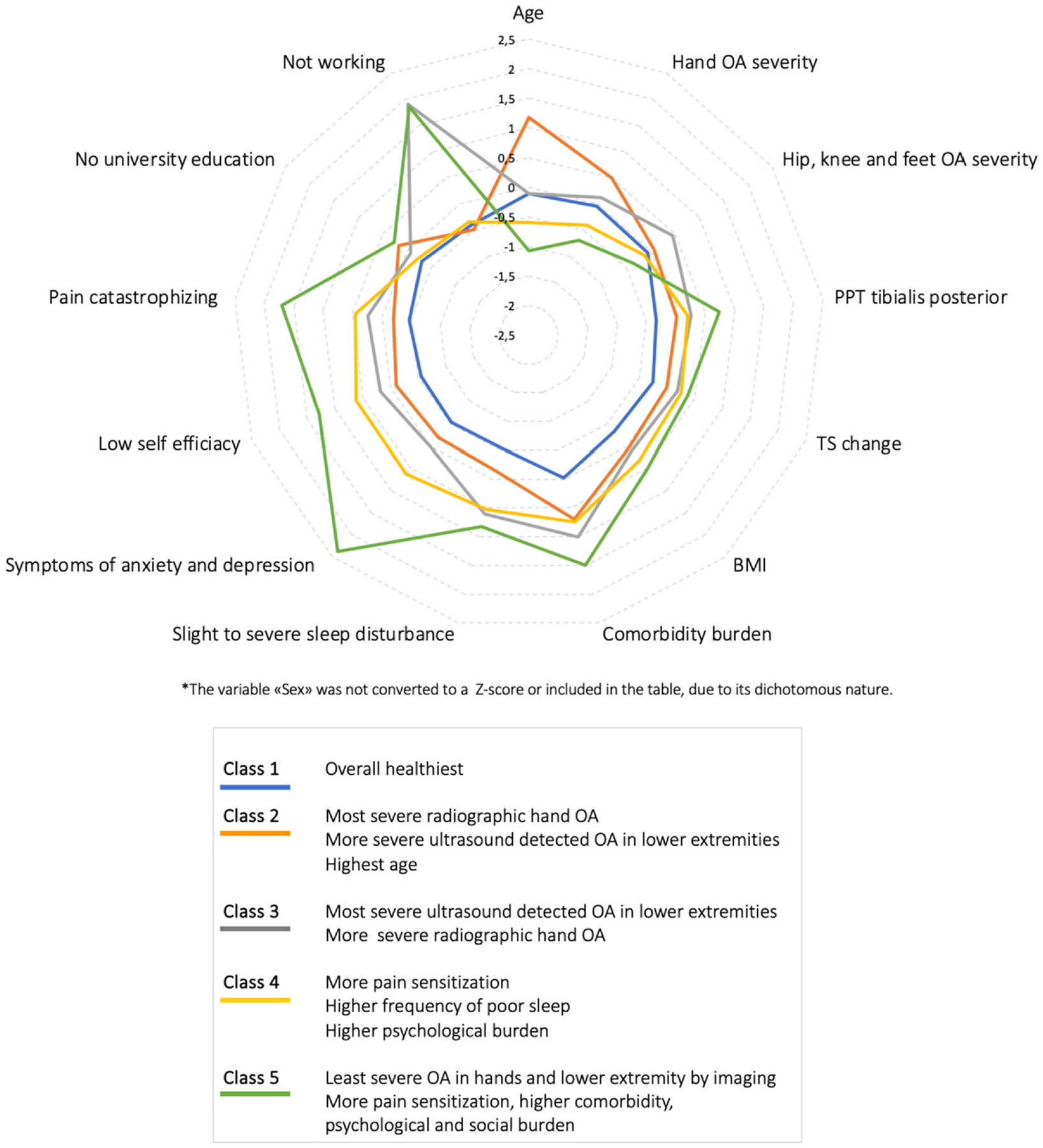
Characteristics of biopsychosocial factors across the proposed hand OA phenotypes. Indicator variables* are presented as Z-scores, which indicates how many standard deviations the data point is below or above the study population mean, where the mean = 0 and one SD= 1. Higher values indicate higher burden.

**Fig. 3:**
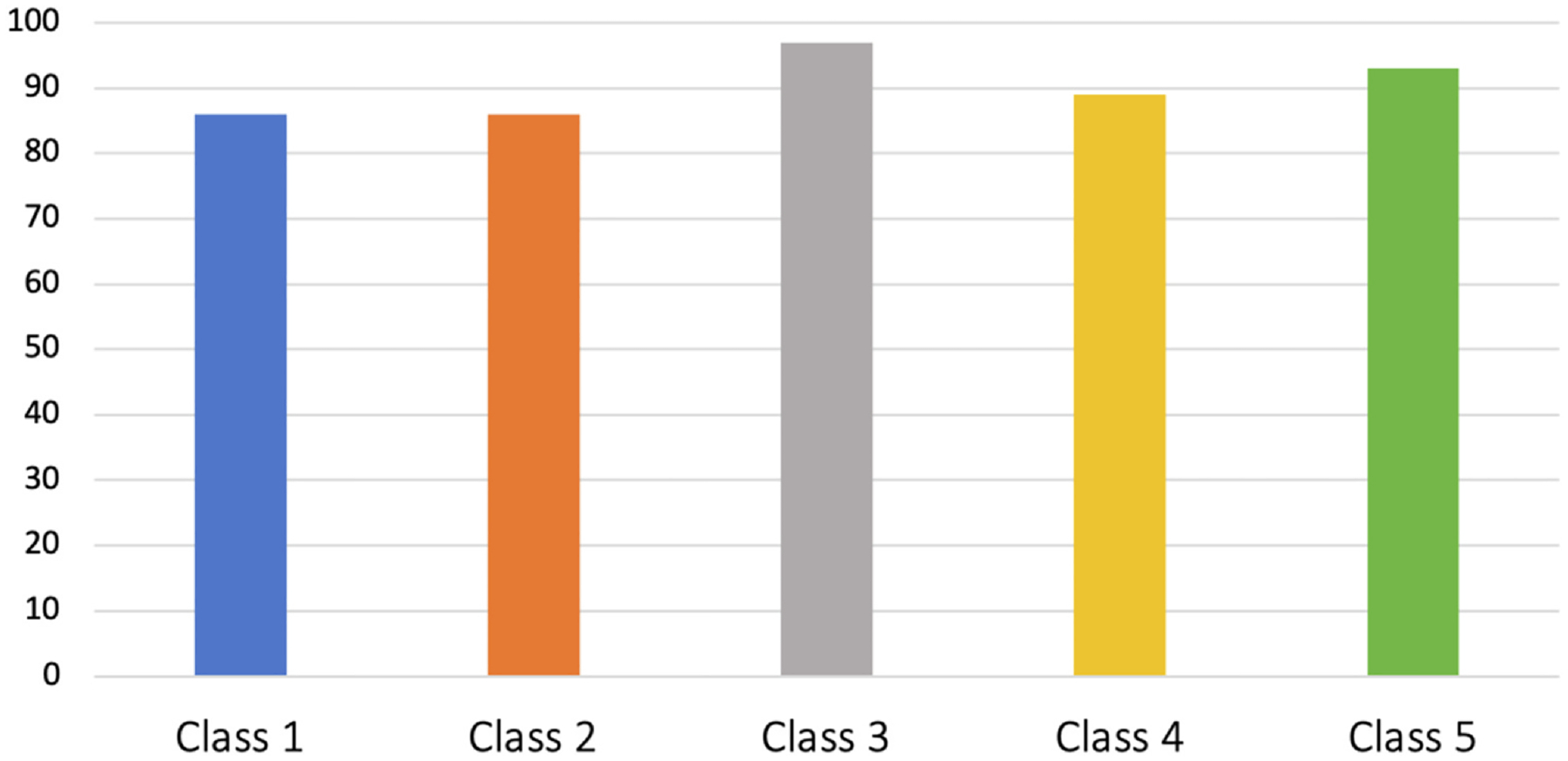
Distribution of women across the hand OA phenotypes. Data presented as percentages (%).

Among the 5 classes (Table I), *Class 1* (n = 110, 37%) was overall the “healthiest” class with low levels of biological, psychological and social burden, and this class had the lowest proportion of women together with Class 2. Classes 2 (n = 70, 23%) and 3 (n = 39, 13%) demonstrated most severe radiographic OA in the hands and ultrasound osteophytes of the lower extremities compared with the other classes. Among these two classes, the hands were most severely affected in class 2 and the lower limbs were most severely affected in Class 3. The two classes differed in age (highest in Class 2) and work participation (no working participants in Class 3). Class 4 (n = 66, 22%) and in particular Class 5 (n = 15, 5%) demonstrated more severe pain sensitization, higher BMI and sleep problems than the other classes. In addition, Class 5 had the most psychological distress and the lowest frequencies of participants with a university education. Although class 5 was of younger age, none of the members of this class where currently working. Further differences between the classes are detailed in Table I, Figs. 2 and 3.

### Pain intensity across the proposed hand OA phenotypes

**Fig. 4:**
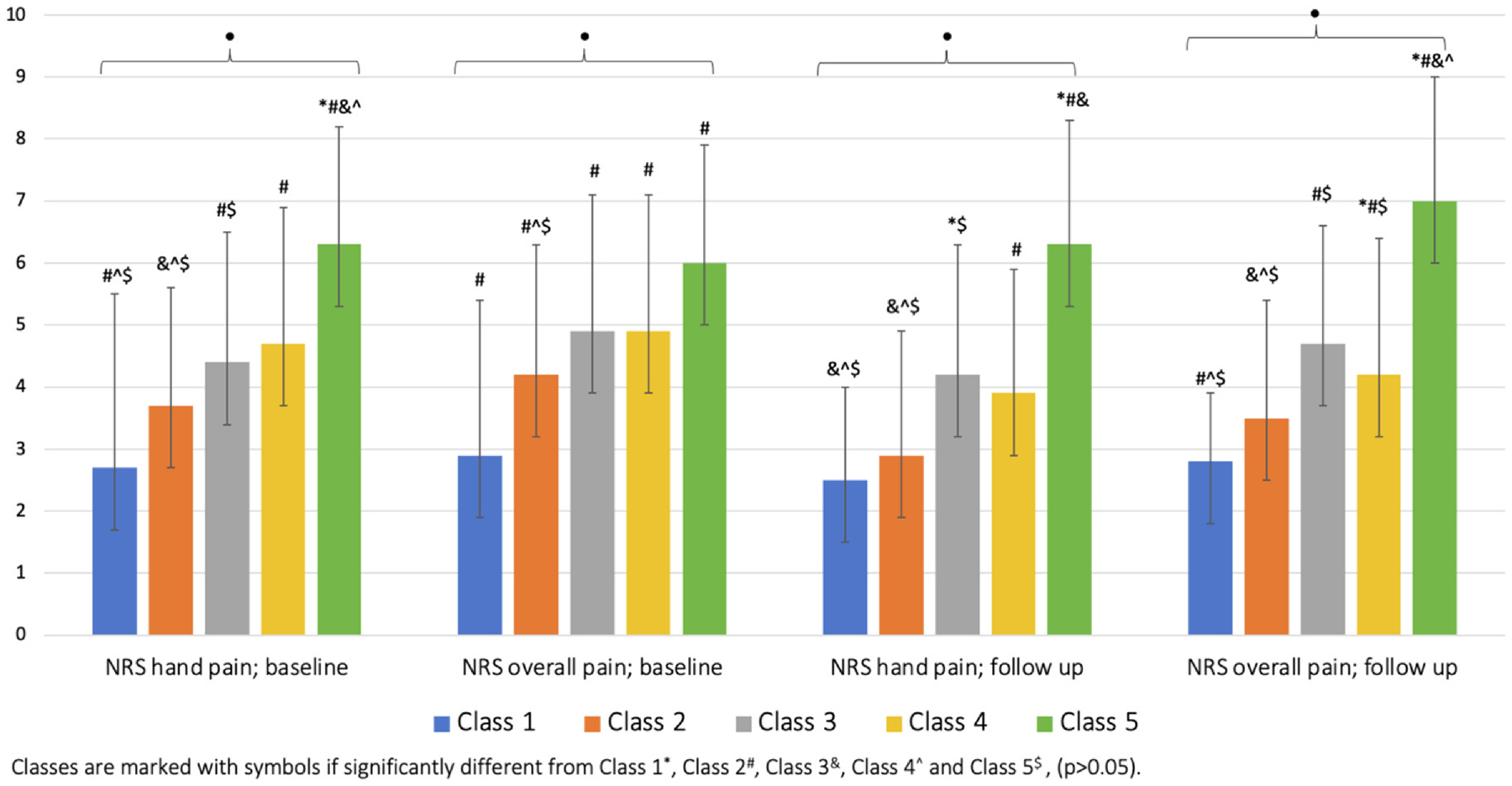
Differences in pain intensity across the hand OA phenotypes. Data presented as mean (SD). * Significant difference between the classes, p < 0.001.

Hand and overall bodily pain differed significantly between the five classes (Fig. 4). Class 1 reported the lowest levels of hand pain at baseline (mean (SD): 2.7 (1.9) and follow-up (mean (SD): 2.5 (2.0)), whilst Class 5 reported the highest levels of pain at baseline (mean (SD): 6.3 (2.8)) and follow-up (mean (SD): 6.3 (1.5)). The results were similar for NRS overall pain. Class 3 reported higher pain levels in overall body than the hands. Overall, there were small changes in pain at follow-up in the hands (mean (SD) −0.4 (2.2)) and overall body (mean (SD): −0.4 (2.4)). Class 5 was significantly different from each of the other classes for pain at baseline and follow-up, except class 3 and 4 for NRS overall pain at follow-up. Differences are detailed in Fig. 4, and in Supplementary Table III.

The associations between classes and pain outcomes are presented in Table II. Significant associations between classes and severity of hand and overall bodily pain were found at baseline and follow-up (Table II). Class 2, 3, 4 and 5 had statistically significantly more pain in overall body than Class 1 with the strongest association observed for Class 5. Similar results were found for hand pain with the exception of Class 2 (i.e. not statistically significantly higher pain in comparison with class 1 at baseline and follow-up). In comparison with class 1, people in Class 5 reported more than 3 points higher NRS pain in hands (beta=3.65, 95% CI 2.53, 4.75) and overall body (beta=3.08, 95% CI 1.95, 4.22) than those in class 1. Class 4 was the only class with a statistically significant association with reduction in overall pain (beta=−0.93, 95% CI −1.79, −0.07) and a borderline statistically association with reduction in hand pain (beta=−0.75, 95% CI −1.55, 0.05) at follow-up.

## Discussion

This study is the first to explore phenotypes in hand OA based on a biopsychosocial model of pain, and their relationship with pain and change in pain during a 3.5-year-follow-up period. We found five distinct possible phenotypes, which differed with regards to biopsychosocial factors and OA severity. The phenotypes also differed in their severity of pain in hands and overall body at baseline and 3.5 years later. In general, the phenotypes that were characterized by lower OA imaging severity (class 4 and 5), but more pain sensitization, higher BMI, poor sleep, pain catastrophizing, and psychological burden reported more pain than the phenotypes with higher OA severity of hands and lower extremities (class 2 and 3) and the overall healthiest class (class 1).

Most previous research in this area has focused on knee OA. Factors representing biological, psychological and social domains, such as multimorbidity, increased pain sensitivity, poor sleep, pain catastrophizing and psychological distress have been found prominent in potential phenotypes with increased pain levels across several knee OA studies.^25–29^ A systematic review aiming to summarize distinct sets of variables represented in clinical knee OA phenotypes found evidence to support a chronic pain phenotype.^30^ This phenotype was distinguished by increased pain sensitization by lower pressure pain threshold and increased temporal summation, as well as presence of factors such as psychological distress, poor coping style and sleep disturbance. The results of our study echo these findings, in which the class with the least OA imaging severity and more presence of psychological distress, pain catastrophizing, and pain sensitization (Class 5) reporting the most pain. Additionally, two other classes identified in this study (Classes 3 and 4) were differentiated by one class mainly characterized by higher OA imaging severity in hands and lower extremities (class 3), whilst the other being characterized by low OA severity but higher biopsychosocial burden (class 4). These two classes reported similar levels of pain, indicating different drivers of pain. Carlesso et al. found in one cross-sectional study that the characteristics of female sex, younger age, lower levels of optimism and self-efficacy as well as measures of pain sensitization (temporal summation) did significantly differ between the phenotypes in a knee OA population going to a first-time orthopedic consultation.^28^ Whilst, in another study by Carlesso et al. including participants without persistent knee pain from the longitudinal Multicenter Osteoarthritis (MOST) Study cohort reported that person-related factors such as poor sleep and psychological factors did not differ substantially between the classes, whilst the four identified phenotypes seemed to be determined by measures of pain sensitization (QST).^29^ Beyond the choice of indicator variables, the difference between the findings in the studies indicate that the phenotypes are dependent on disease progression and symptoms.^28^ Thus, certain factors or interaction of factors may be at play at different stages of the disease progression. It is a challenge to compare the existing literature as the choices of study population, statistical methods, defining variables and measuring methods vary greatly. The phenotypes are *modal* latent classes. Membership to a latent class is based on the most likely class, and there is no uniformly predicted latent class. However, it appears that phenotype characteristics found in our study such as increased pain sensitization, higher psychological distress and multimorbidity including poor sleep, align with previous studies.

Knowledge regarding knee OA phenotypes and pain may be transferrable to other joints, even if risk factors for pain may vary across joint sites.^31^ It has been suggested that systemic processes are more important risk factors for hand OA than for weight-bearing joints, such as knee OA,^32^ and the drivers of pain may thus differ. Evidence from studies support a relationship between factors of metabolic syndrome and OA pain.^33,34^ A previous study including the same cohort, found that higher BMI was associated with more pain in the hands, as well as the knees and hips. Findings also indicated that systemic effects due to obesity mediated the relationship between higher BMI and hand pain to a larger degree than for pain in the lower extremities.^8^ Class 5 had higher BMI than the remaining classes, which may reflect a systemic metabolic component. Although phenotypes in hand OA are explored to a limited extent, there are studies in recent years which have focused on establishing phenotypes based on pathological joint changes.^13,14^ Phenotypes in hand OA based on factors beyond the joint has not been previously explored.

The phenotypes differed significantly in terms of pain levels at baseline and 3.5 years later. Participants in the phenotype with lower hand OA radiographic severity, but more poor sleep, higher levels of pain sensitization, higher BMI, pain catastrophizing, comorbidity and psychological burden (class 5) reported the highest level of pain. These results support the previous findings on the importance of pain sensitization and psychological factors in the hand OA pain experience.^7,10,35–37^ Due to the recruitment of participants from a rheumatology outpatient clinic, we cannot exclude the possibility that patients are being referred from primary care due to severe pain despite little objective findings of OA, which may contribute to stronger associations. The association between the phenotype with the most hand OA imaging severity (class 2) and self-reported hand pain at the follow-up examination did not reach statistical significance, likely due to the lower levels of pain reported.

The changes in overall pain were generally small, and most classes tended to have less pain at follow-up than at baseline (except class 5 who had on average a small increase in overall pain). Hence, we were not able to identify predictors of pain progression in this study. The high levels of pain at the baseline visit may be explained by a disease flare, which led to referral of the patient to secondary care and thereafter inclusion in the Nor-Hand study. However, the changes in pain may also be a reflection of and driven by changes in the indicator biopsychosocial themselves. Furthermore, pain in hand OA is often fluctuating, and we were unable to capture measures of fluctuations in pain with only two time points with assessment of pain.

Our results indicate the combination of more pain sensitization, poor sleep, higher comorbidity, BMI, psychological and social burden may exacerbate current and long-term pain in people with hand OA. This underlines the importance of addressing the pain experience in patients with hand OA. The novelty of this study is the exploring of biopsychosocial factors in combination with each other, rather than individually, and how these phenotypes are related to the pain experience. The differences in these hand OA phenotypes highlights the different contributions to the pain experience in people with hand OA and speaks to the need to personalize/tailor treatments to those underlying contributors rather than simply applying a one-size-fits-all approach. The possible phenotypes which are more burdened by the biopsychosocial factors (class 4 and 5) may be representative of people in the hand OA population with an increased need for a multimodal treatment approach. However, class 5 was also the smallest class in the model which may impact the generalizability of this class, which should be accounted for when interpreting the results. A similar phenotype with higher biopsychosocial burden was found in patients with common musculoskeletal complaints, and this group was also the smallest of the five identified phenotypes. This study had a larger sample (N = 435), however the phenotype was of larger size (11%) relative to the remaining phenotypes in comparison to the phenotype (5%) in our study.^38^ Recommendations have stated that classes should be no less than 5%, although this recommendation has become less rigid.^23^ Other studies have reported phenotypes at this threshold of size, as well as lower in cases of larger study samples.^39^ The analyses in this study are exploratory and the classes are modal, and the results would need to be validated in other cohorts. However, addressing modifiable factors, such as multimorbidity, sleep, pain catastrophizing and self-efficacy where indicated, may likely benefit the patient in a clinical setting with regard to their hand OA pain experience.

The Nor-Hand cohort represents the first study on hand OA using the biopsychosocial framework to define phenotypes and explore their relation to pain using longitudinal data. We included study participants with a wide range of symptoms, which may have increased the generalizability of our results, even if the generalizability may be limited by the overrepresentation of people with higher education, good physical and mental health, high proportion of women and that the patients were referred to specialized health care. There are other limitations to this study. A limitation to consider when interpreting the results is that the complex relationship between variables may be oversimplified which may influence the class memberships, due to the one step approach of our LCA analysis. There was a 27% loss to follow-up in this study, but these participants displayed similar characteristics as the participants included in the analyses (Supplementary Table I). The outbreak of the corona pandemic in 2020 was during the follow-up data collection, which may have impacted the willingness to participate further in the study. We included measures of education and work status as a proxy for socioeconomic status. Work status is likely a less sensitive measure for socioeconomic status, as it does not assess directly assess the individual’s economic status and should be interpreted accordingly. We also acknowledge that it would be of interest to include a measure of symptom duration, as that may have been a contributor in distinguishing the possible phenotypes. However, an adequate measure of symptom duration was not available in this dataset. With regard to quantitative sensory testing, the inter-reader reliability was lower than in previous studies^42,43^. We repeated the statistical analyses on data that were only collected by the examiner who performed the majority of the assessments. The results were similar to those described, which indicates that the present results were not influenced by the moderate inter-observer reliability. In our study we have tested TS at only one site, which may be close to pathology. We acknowledge that this site might have been influenced by peripheral sensitization.

## Conclusion

To summarize, we found five potential phenotypes of hand OA using a biopsychosocial framework. There were significant differences in pain intensity between patients belonging to the different phenotypes. The phenotype with the least radiographic hand OA severity, but more pain sensitization, comorbidities, higher BMI, psychological and social burden, reported more pain in hands or overall body than the phenotype with the most severe OA imaging in the hands and in lower extremities. This is a possible reflection of the structure-symptom discrepancy in hand OA. Being able to identify possible modifiable risk factors for pain and adjust the treatment accordingly is of clinical relevance.

## Supplementary Material

supplemental materials

## Figures and Tables

**Table I:** Characteristics of the biopsychosocial variables across the proposed hand OA phenotypes.

	Class 1 N = 110, 37%	Class 2 N = 70, 23%	Class 3 N = 39, 13%	Class 4 N = 66, 22%	Class 5 N = 15, 5%
*Biological domain:*					
Age	60 (6)*	68 (2)^^^	60 (5)^♯^	57 (5)^•^	54 (5)◊
Sex, n (%) women	95 (86)*	60 (86)^^^	38 (97)^♯^	59 (89)^•^	14 (93)
Radiographic OA severity (KL, 0–128)	29.4 (16.7)*	39.9 (19.9)^^^	32.6 (20.7)^♯^	22.6 (17.4)^•^	17.1 (15.4)◊
Hip, knee and feet OA (Ultrasound score 0–18)	4.4 (3.3)*	4.8 (3.2)^^^	6.1 (3.7)^♯^	4.2 (3.1)^•^	3.4 (2.7)◊
PPT tibialis anterior	6.4 (2.6)*	5.5 (2.8)^^^	4.9 (2.4)^♯^	5.0 (2.1)^•^	3.6 (1.7)◊
TS change	1.2 (1.4)*	1.6 (1.5)^^^	1.9 (1.7)^♯^	2.0 (1.7)^•^	2.2 (1.5)◊
BMI	24.9 (3.5)*	26.7 (5.7)^^^	27.1 (4.6)^♯^	28.1 (5.6)^•^	29.1 (5.5)◊
Comorbidity burden (0–42)	5.6 (3.3)*	8.4 (4.5)^^^	9.6 (3.5)^♯^	8.6 (3.8)^•^	11.6 (5.3)◊
Slight to severe sleep disturbance, n (%)	61 (56)*	48 (70)^^^	35(90)^♯^	64 (97)^•^	15 (100)◊
*Psychological domain:*					
HADS (0–42)	4.0 (3.5)*	6.1 (5.2)^^^	7.3 (4.5)^♯^	11.1 (5.3)^•^	21.8 (5.8)◊
ASES (10–100)	76.2 (11.1)*	69.5 (15.5)^^^	65.6 (10.2)^♯^	59.1 (11.5)^•^	49.5 (15.1)◊
PCS (0–58)	7.1 (5.9)*	9.3 (6.1)^^^	12.9 (8.7)^♯^	14.6 (7.5)^•^	24.8 (8.7)◊
*Social domain:*					
University education, n (%)	78 (72)*	36 (51)^^^	20(51)^♯^	34 (52)^•^	6(40)◊
Working, n (%)	103 (96)*	67 (96)^^^	0 (0)^♯^	56 (85)^•^	0 (0)◊

Classes with different symbols are significantly different at p < .05 per indicator variable. Data presented as mean (SD) unless otherwise indicated.

SD, standard deviation; OA, osteoarthritis; KL, Kellgren-Lawrence; PPT, pressure pain threshold; TS, temporal summation; BMI, body mass index; HADS, hospital anxiety and depression scale; PCS, pain catastrophizing scale; ASES, arthritis self efficacy scale.

**Table II:** Associations of hand OA phenotypes to pain outcomes.

	Baseline NRS pain		Follow-up NRS pain		Change in NRS pain	
	Hands	Overall body	Hands	Overall body	Hands	Overall body
Class 1	Ref.	Ref.	Ref	Ref	Ref	Ref
Class 2	**1.03 (0.41, 1.65)**	**1.24 (0.61, 1.87)**	0.38 (−0.34, 1.09)	**0.71 (0.01, 1.43)**	−0.48 (−1.27, 0.33)	−0.52 (−1.39, 0.35)
Class 3	**1.70 (0.94, 2.45)**	**1.95 (1.18, 2.72)**	**1.66 (0.79, 2.52)**	**1.88 (1.01, 2.76)**	−0.13 (−1.10, 0.84)	−0.11 (−1.18, 0.95)
Class 4	**1.97 (1.34, 2.61)**	**1.95 (1.31, 2.59)**	**1.33 (0.62, 2.04)**	**1.35 (0.63, 2.06)**	−0.75 (−1.55, 0.05)	**−0.93 (−1.79, −0.07)**
Class 5	**3.65 (2.53, 4.75)**	**3.08 (1.95, 4.22)**	**3.72 (2.28, 5.14)**	**4.19 (2.74, 5.64)**	−0.11 (−1.71, 1.49)	0.67 (−1.06, 2.40)

Bold indicates statistically significant associations. Data presented as beta (95% CI).

CI, confidence interval; NRS, numeric rating scale.
