# Supplementary material for: Hand osteoarthritis phenotypes based on a biopsychosocial approach, and their associations with cross-sectional and longitudinal pain
Source: Osteoarthritis Cartilage. Author manuscript; Available in PMC 2025 Aug 1. (PMC11254542; doi:10.1016/j.joca.2024.04.011)
Supplement: supplemental materials [file NIHMS2001312-supplement-supplemental_materials.pdf]

Supplementary material

Supplementary table 1:
Characteristics of the baseline population and the population lost to follow-up

	Baseline population N= 300	*Lost to follow-up N=87*
Age	60 (6)	60 (7)
Sex, n (%) women	266 (88)	78 (93)
Radiographic OA severity (KL, 0-128)	30.2 (19.2)	28.5 (19.9)
Hip, knee and feet OA (ultrasound score 0-18)	4.6 (3.3)	4.1 (2.9)
PPT tibialis anterior	5.5 (2.5)	5.4 (2.2)
TS change	1.6 (1.6)	1.7 (1.6)
BMI	26.5 (5.0)	26.3 (5.5)
Comorbidity burden (0-42)	5.6 (4.0)	6.0 (4.1)
Slight to severe sleep disturbance, n (%)	225 (75)	66 (76)
HADS (0-42)	7.3 (6.2)	8.1 (6.9)
ASES (10-100)	68. 5 (14.1)	66.6 (13.8)
PCS (0-58)	11.0 (8.2)	11.1 (8.7)
University education, n (%)	174 (58)	44 (50)
Working, n (%)	153 (53)	36 (40)
NRS hand pain, mean (SD) [0-10]	3.8 (2.3)	3.9 (2.3)
NRS all joint pain, mean (SD) [0-10]	4.1 (2.3)	4.2 (2.2)

Supplementary table 2:
Model of fit criteria for the models with 3-6 classes

Models	AIC	BIC	Likelihood bootstrap test
Class 3	14868.65	15087.17	-7832.231
Class 4	15017.06	15231.88	-7745.069
Class 5	14621.91	14929.32	-7679.157
Class 6	9648.772	702.553	-7629.014

Supplementary table 3:
Differences between each class by NRS pain

	Class	Classes	P-value
	**Baseline**		
NRS hand pain	1	2	0.01
		3	0.07
		4	1.00
		5	>0.001
	2	1	0.01
		3	>0.001
		4	>0.001
		5	>0.001
	3	1	0.08
		2	>0.001
		4	>0.001
		5	>0.001
	4	1	1.00
		2	>0.001
		3	1.00
		5	0.02
	5	1	>0.001
		2	>0.001
		3	0.05
		4	0.02
NRS overall bodily pain	1	2	>0.001
		3	0.49
		4	0.92
		5	0.02
	2	1	0.49
		3	>0.001
		4	>0.001
		5	>0.001
	3	1	0.49
		2	>0.001
		4	1.00
		5	0.58
	4	1	0.92
		2	>0.001
		3	1.00
		5	0.76
	5	1	0.02
		2	>0.001
		3	0.58
		4	0.76
	**Follow-up**		

NRS hand pain	1	2	1.00
		3	0.23
		4	0.09
		5	>0.001
	2	1	0.23
		3	>0.001
		4	>0.001
		5	>0.001
	3	1	0.23
		2	>0.001
		4	1.00
		5	0.02
	4	1	0.09
		2	>0.001
		3	1.00
		5	0.02
	5	1	>0.001
		2	>0.001
		3	0.02
		4	0.10
NRS overall bodily pain	1	2	0.53
		3	1.00
		4	0.19
		5	>0.001
	2	1	1.00
		3	>0.001
		4	>0.001
		5	>0.001
	3	1	1.00
		2	>0.001
		4	1.00
		5	>0.001
	4	1	0.19
		2	>0.001
		3	1.00
		5	0.05
	5	1	>0.001
		2	>0.001
		3	>0.001
		4	0.05
